# An Ensemble Patient Graph Framework for Predictive Modelling from Electronic Health Records and Medical Notes

**DOI:** 10.3390/diagnostics15060756

**Published:** 2025-03-18

**Authors:** S. Daphne, V. Mary Anita Rajam, P. Hemanth, Sundarrajan Dinesh

**Affiliations:** 1 Department of Computer Science and Engineering, CEG Campus, Anna University, Chennai 600025, India; daphnesam7@gmail.com (S.D.); hemanthpalani001@gmail.com (P.H.); dineshsunraj27@gmail.com (S.D.); 2 Centre for Cyber Security, Department of Computer Science and Engineering, CEG Campus, Anna University, Chennai 600025, India

**Keywords:** knowledge graph, graph convolutional network, natural language processing, clinical BERT, BioBERT, BlueBERT

## Abstract

**Objective:** Electronic health records (EHRs) are becoming increasingly important in both academic research and business applications. Recent studies indicate that predictive tasks, such as heart failure detection, perform better when the geometric structure of EHR data, including the relationships between diagnoses and treatments, is considered. However, many EHRs lack essential structural information. This study aims to improve predictive accuracy in healthcare by constructing a Patient Knowledge Graph Ensemble Framework (PKGNN) to analyse ICU patient cohorts and predict mortality and hospital readmission outcomes. **Methods:** This study utilises a cohort of 42,671 patients from the MIMIC-IV dataset to build the PKGNN framework, which consists of three main components: (1) medical note extraction, (2) patient graph construction, and (3) prediction tasks. Advanced Natural Language Processing (NLP) models, including Clinical BERT, BioBERT, and BlueBERT, extract and integrate semantic representations from discharge summaries into a patient knowledge graph. This structured representation is then used to enhance predictive tasks. **Results:** Performance evaluations on the MIMIC-IV dataset indicate that the PKGNN framework outperforms state-of-the-art baseline models in predicting mortality and 30-day hospital readmission. A thorough framework analysis reveals that incorporating patient graph structures improves prediction accuracy. Furthermore, an ensemble model enhances risk prediction performance and identifies crucial clinical indicators. **Conclusions:** This study highlights the importance of leveraging structured knowledge graphs in EHR analysis to improve predictive modelling for critical healthcare outcomes. The PKGNN framework enhances the accuracy of mortality and readmission predictions by integrating advanced NLP techniques with patient graph structures. This work contributes to the literature by advancing knowledge graph-based EHR analysis strategies, ultimately supporting better clinical decision-making and risk assessment.

## 1. Introduction

Predicting patient outcomes [1,2], especially mortality in critical care settings, has long been a priority in medical research. Numerous clinical parameters have been identified as significant predictors. The length of stays in the intensive care unit (ICU) has been associated with severe circumstances such as mechanical ventilation (MV) and psychiatric medication poisoning, in addition to biochemical indicators. Furthermore, early predicting mechanical ventilation duration for patients suffering from acute respiratory distress syndrome (ARDS) can enhance risk stratification and improve care strategies [3].

Predictive models for assessing risks in critically ill patients, such as hypoglycemia in septic patients or post-surgical outcomes following coronary artery bypass grafting (CABG), are increasingly being explored [4,5]. While CABG remains a critical intervention for patients with coronary atherosclerosis, the long-term prognosis remains uncertain, making the development of predictive models essential for improving patient survival probabilities.

Anyway, the complexity of disorders like *pulmonary hypertension (pH)* emphasises the requirement for all-encompassing prediction models. Despite advancements in therapy, the pathophysiology [6] of pH includes a mix of musculoskeletal, cardiovascular, and respiratory problems that lead to increasing exercise intolerance and a reduced quality of life. Because these comorbidities are multi-dimensional, predictive models that leverage electronic health record (EHR) data are crucial for accurately predicting patient mortality and guiding clinical decision-making.

These results demonstrate the importance of using EHR data to forecast patient mortality. EHRs capture a wide range of clinical information, including biochemical markers, comorbidities, and intervention outcomes. These are key for developing robust mortality risk prediction models to improve patient care and outcomes.

**Figure 1:**
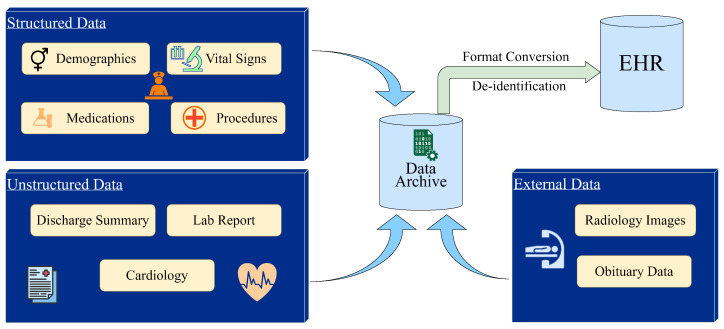
Electronic health records information extraction.

Hospitals often record patient data as EHRs, which include data on tests, symptoms, diagnoses, and prescriptions. These EHRs contain structured patient information, lab report details, and unstructured data in free text comments, such as medical notes. This rich information within EHRs is crucial to integrating knowledge about illnesses, treatments, and proteomics into clinical knowledge graphs, all within a real-time patient care system. Figure 1 shows some example tasks involved in EHR information extraction using a Clinical Data Warehouse (CDW) approach.

Integrating structured and unstructured data in EHRs allows for a comprehensive view of patient health, vital for personalised medicine. Structured data, such as lab results and medication lists, provide clear, quantitative insights. In contrast, unstructured data, like medical notes, offer contextual information and detailed narratives about the patient’s condition and treatment responses. Despite several deep learning methodologies that provide patient-specific death forecasts from unstructured data in EHRs, these current techniques frequently fail to completely extract the concealed, intricate information essential for thorough analysis. Knowledge graphs offer a robust solution by organising unstructured data into interconnected, semantic relationships. They capture complex associations between medical entities—such as symptoms, treatments, and diagnoses—allowing for a more holistic understanding of patient history. By transforming fragmented narratives into a structured form, knowledge graphs enhance the interpretability of unstructured data and enable more accurate predictions, decision-making, and personalised care. This approach bridges the gap between raw clinical narratives and actionable insights, significantly advancing the precision of healthcare analytics.

Knowledge graphs are essential for describing the complex connections and meanings inherent in the data domain. They encapsulate a wide range of biomedical entities and their interrelations, such as diseases, symptoms, drugs, and genes. Using a patient graph network, the framework enables the extraction of meaningful embeddings from the knowledge graph, facilitating the identification of subtle patterns and associations within biomedical information. For instance, a knowledge graph can reveal how specific genetic markers correlate with disease susceptibility or how drug combinations impact patient outcomes.

One effective strategy is to employ GCN for the knowledge graph. These networks leverage data statistics to guide the process of structural learning, presenting a promising approach to unravel the underlying structure inherent in EHR data [7]. GCNs can capture the dependencies and interactions between different features in the data, which traditional flat models might overlook.

**Figure 2:**
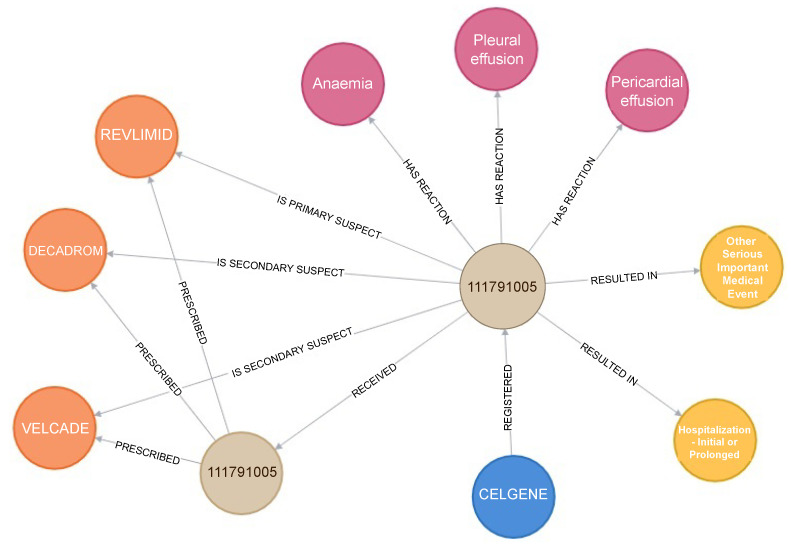
Graphical structure of EHRs using Neo4j [8].

Creating a knowledge graph from EHR data is a collaborative, multidisciplinary effort involving experts in healthcare, data engineering, natural language processing, machine learning, and graph databases. The resulting knowledge graph becomes a powerful tool for healthcare professionals to improve patient care, conduct research, and make more informed clinical decisions. The most common technique for applying neural networks to handle EHR data has been to treat each case as an unordered set of characteristics, essentially representing it as a “bag of features”. Unfortunately, this method disregards the vital geometric structure representing the physician’s assessment process. For instance, when we analyse the encounter in Figure 2 as a bag of features, we lose crucial information that the combination of Decadron, Revlimid, and Velcade drugs prescribed to patient ‘111791005’ was the suspected cause of anaemia, resulting in severe medical conditions.

**Problem Statement**: To predict patient mortality using EHRs, a patient knowledge graph focusing on extracting relationships between entities like diagnoses and treatments from unstructured medical notes for better interpretability and decision support.

A patient network is effectively modelled as a graph, where each node represents an individual patient’s hospital stay, encoded using graph representations derived from their medical notes and medical data. The edges between nodes indicate a connection between two hospital stays based on a similarity measure, such as shared diagnoses, treatment responses, or other medical characteristics. The objective of this model is to be on par with the decision-making process of healthcare professionals. In clinical practice, doctors rely on a patient’s medical history and draw on their experience with patients who have exhibited similar conditions or treatment responses. Using this graph-based approach, we can simulate this process computationally, allowing the model to inform decisions about medication, treatment plans, or interventions by identifying patterns and outcomes from similar patients. This knowledge graph captures the implicit knowledge gained from prior cases, supporting personalised and evidence-based care recommendations. This knowledge graph is the input for GCN, which learns the patient embeddings from the patient graph, encouraging a regularised latent space for the embeddings.

Thus, to address the problems of EHR data that does not always provide complete structure information, we propose PKGNN, an ensemble approach for concurrently learning the hidden structural information for different prediction tasks. We contribute the following in this paper:

- The PKGNN (Patient Knowledge Graph Framework) model is proposed as a concrete design and convincing implementation for biomedical data. It integrates patient graph topology and health record data to enhance two tasks: mortality prediction and 30-day hospital readmission prediction.
- An ensemble model is implemented to learn the underlying EHR structure using a graph convolutional network (GCN). The data are pre-trained on three different clinical models: Clinical BERT [9], BioBERT [10], and BlueBERT [11], depicting a promising method for binary classification.
- We validate the proposed PKGNN on the openly accessible EHR database, MIMIC-IV [12].

This study uses the MIMIC-IV benchmark dataset to compare the performance of the proposed framework with that of SOTA deep learning models and predict critical patient outcomes. The models’ performances have been evaluated for mortality and 30-day hospital readmission predictions.

## 2. Literature Survey

Large-scale, balanced training data are frequently necessary for deep learning (DL) models in the healthcare industry to be reliable, flexible, and effective. Creating DL models has been significantly tricky when the data are hugely imbalanced. Healthcare providers face difficulties caring for elderly people due to increased complexity and comorbidities [13]. These challenges include balancing beneficial and detrimental therapies, keeping an eye on declining patients, and allocating resources. Mortality prediction makes decision-making quicker [14]. This study aims to emphasise the current challenges in risk prediction for the prevalence of 30-day hospital readmission and analyse its impact on mortality in ICU patients.

### 2.1. Latent Embeddings for Medical Notes

Medical notes contain detailed patient information, including symptoms, diagnosis, radiological results, daily activities, and sickness history. Medical notes provide vital information, but identifying trends can be tricky [9]. Their writing uses unconventional vocabulary and acronyms, and their styles are varied [15]. The high dimensionality and sparseness of healthcare records have led to a lot of research on building prediction models utilising structured EHR data elements [16,17].

Due to developments in deep learning algorithms, extracting crucial clinical information from medical records using transfer learning and contextual word embedding models has gained momentum. Bidirectional Encoder Representations from Transformers (BERT) [18] is a contextualised text representation model based on a masked language model pre-trained on a large clinical text corpus using the bidirectional transformer encoder architecture. These embeddings are then used in downstream tasks. Clinical prediction models often integrate BERT design into downstream tasks and fine-tune it to provide integrated task-specific architecture [9,10,19].

Clinical BERT [9], introduced by Huang et al., is a specialised adaptation of the BERT model explicitly designed for the medical and clinical domain. Standard NLP models like BERT are pre-trained on general language corpora, which may not capture the fine distinction and specific terminology used in medical texts. Clinical BERT has a vocabulary that is fine-tuned by a large corpus of medical notes from the MIMIC-III dataset to include medical terms, which improves its understanding of clinical texts compared to general-purpose models. Leveraging the BERT architecture, Clinical BERT captures the context of words bidirectionally, meaning it considers the entire sentence before assigning meaning to a word. This is particularly useful in clinical texts, where context is critical.

BioBERT is initialised with the original BERT weights and then pre-trained on full-text PubMed Central articles and PubMed abstracts. While Clinical BERT, BioBERT, and BlueBERT are based on the BERT architecture and use medical notes from the MIMIC-III dataset for pre-training, there are significant variations between the models. One crucial distinction in their training corpora is that Clinical BERT is pre-trained only on medical notes, whereas BlueBERT is pre-trained on medical notes of MIMIC-III and PubMed abstracts. DeepNote-GNN [20], introduced by Golmaei et al., employs a deep learning model that combines a pre-trained BERT with a patient graph to predict hospital readmissions.

### 2.2. Learning Graph Representation on EHR

Graph Convolutional Networks have the capacity to learn about node attributes as well as graph structure. They might be accomplished by using semi-supervised learning methods for classifying nodes.

For specific graph learning objectives, graph representation aims to learn a feature vector for a subset or an entire graph. The graphs generated from EHRs are frequently heterogeneous since they often comprise many healthcare entities and several relations. Consequently, heterogeneous graphs may not be directly fitted with the GCN.

GCNs use message-passing techniques to combine information from neighbouring nodes, allowing them to capture both local and global dependencies in graph-structured data, as shown by Wu et al. [21]. This makes GCNs particularly suitable for applications such as gene property prediction, social media analysis, and knowledge graph reasoning, where understanding relationships is critical.

Recent advancements in GNNs have also focused on their ability to learn hierarchical representations through multi-layer architectures, as highlighted by Liu et al. [22]. Their research showed that GNNs are an effective method for tasks like graph and node categorisation because they can accurately simulate intricate structural patterns. These properties, combined with their scalability and adaptability to dynamic graphs, underscore the growing importance of GNNs in analysing complex systems.

For disease prediction, Sun et al. [23] created two dual graph networks (bipartite) with two kinds of nodes each: a patient record and a medical concept graph. Rather than using the node categories in the propagation rule, they used the projection weight to bring all node embeddings onto a shared space. MedGCN [24] employs GCN and trains the model with a cross-regularisation strategy for medication recommendation. It constructs a bipartite subgraph between lab test information and encounters. One task’s loss might be considered a regularisation term for another task in cross-regularisation.

From various EHR data, MGATRx [25] created a graph with six different node categories (medication, sickness, the desired level, the base, adverse effect, and connect). It then extended multi-view max pooling for drug repositioning using an attention mechanism. The MedGCN graph is composed of four types of nodes: patients, medications, vitals, and diagnosis. It is used for drug recommendation and laboratory task imputation.

Instead of using GCN spectrum filters, Graph Attention Network (GAT) [26] compares each node in the network with its closest neighbours to learn regional characteristics. It can learn graph topologies from attention variables and may design various weights to edges, increasing the model’s ability and interpretation.

Each patient’s medical encounter includes a hospital stay embedding combined with the medical notion embeddings introduced in Graph Convolution Transformer (GCT) [7]. The challenge of uniformly distributed attention weights among medical ideas is resolved by GCT by regularising a pre-established graph. The construction of GCT involves linking several categories of medical ideas (like treatment, laboratory, and procedures) in order to replicate doctors’ decision-making processes.

The clinical prediction models built with these standard architectures have a few limitations: The fine-tuning strategy adds few task-specific factors, has limited generalisation capability, and fails to recognise the extended interdependence of words, which are critical in clinical situations. However, specially designed approaches restrict the generalisation of machine learning models. Pre-compiling a costly representation of training information and running trials with less expensive task-specific classification models has significant computational benefits [18].

In summary, although these studies have achieved satisfactory learning structures, the absence of pre-established graphs is still challenging. Thus, we solve the limitations mentioned above by introducing the fusion of a deep learning model that uses medical notes and a knowledge network for prediction. This study uses feature aggregation to improve the depiction of medical notes. The medical knowledge graph can potentially capture more meaningful and robust representations of medical concepts and their relationships. These representations can be helpful for various tasks, including disease prediction, drug discovery, and patient cohort analysis.

## 3. Materials and Methods

This section defines the proposed PKGNN, focusing on clinical risk prediction problems with EHR data. The proposed ensemble GCN architecture utilises medical notes [27,28] with feature extraction using pretrained BERT variant models.

### 3.1. Datasets

We validate the proposed PKGNN on a real-world EHR database, Medical Information Mart for Intensive Care (MIMIC-IV) [29], which is openly accessible. We selected the following two forecasting tasks to evaluate the performance of the proposed models.

*The 30-day Hospital Readmission* is a binary classification task that aims to predict whether a patient, at time *t*, will have to be re-admitted to the hospital in the next 30 days. We evaluate the AUROC and AUPRC metrics.

*Mortality prediction* is a binary classification task that aims to predict whether a patient, at time *t*, will expire in the upcoming 24 h. We evaluate the AUROC and AUPRC metrics.

The MIMIC-IV [12] and MIMIC-IV discharge summary notes [27] database undergo a selection process to identify a subset of data records for our patient cohort, omitting irrelevant and redundant features. The cohort comprises individuals aged 18 years or older who have spent a minimum of one day in the ICU, with an average daily duration exceeding six hours. Patients who are not organ donors and have not been transferred from another hospital are included in our cohort. To minimise ambiguity, we exclude individuals with conditions such as neuromuscular diseases, malignant tumours, and severe burns, which typically require extended hospital stays. Every ICU stay record includes both time-series and static characteristics (e.g., age, gender).

**Figure 3:**
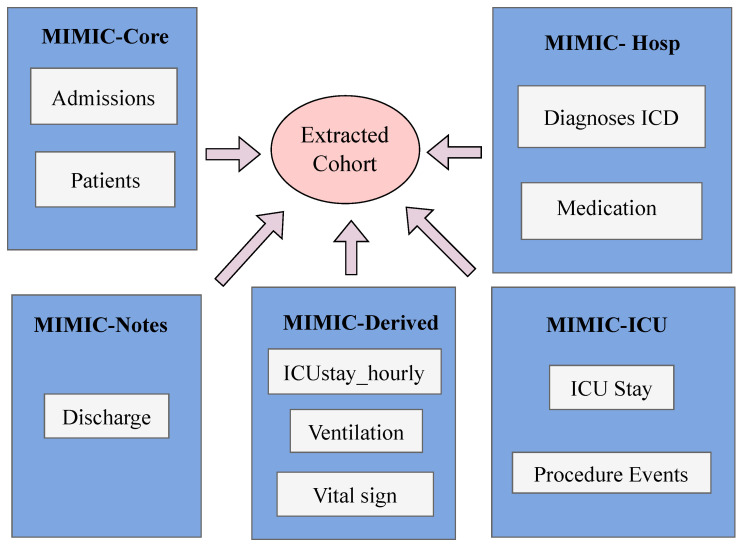
Data extraction from MIMIC-IV tables.

Figure 3 summarises the cohort data that were taken from the tables in the MIMIC-IV database. The 35 unique tables that make up the MIMIC-IV relational database are divided into four different modules that correspond to the core, hospital, intensive care unit, and derived tables. We extract information from the admissions, patients, and icu_stay tables according to the cohort requirements. Further, we transfer the ICD diagnostic codes to the cohort selection schema by mapping them from the diagnosis_icd table. Accessing the derived tables, ICUstay_hourly and vitalsign, is necessary to retrieve the hourly details of patients and their routines. Then, the discharge table’s discharge summary text field is concatenated to create the final complete cohort.

### 3.2. Problem Formulation

**Figure 4:**
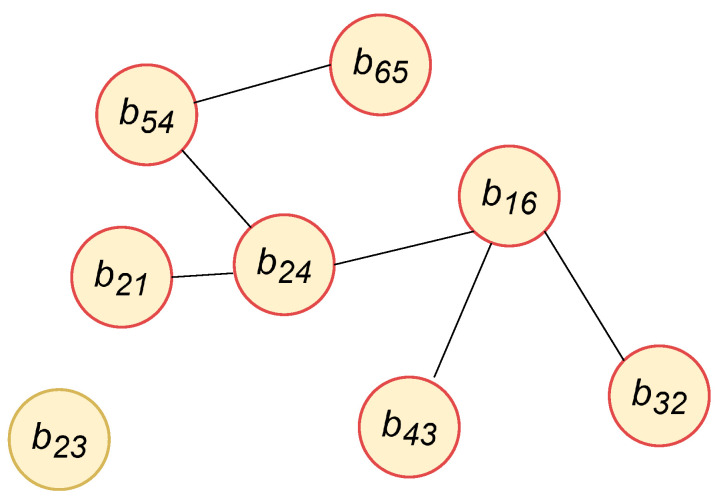
An example of a patient knowledge graph, where the node ($b_{23}$) serves as an illustration of the node without any edge, which means hospital stays, $b_{23}$ has no similar hospital stay features in the network. All the other nodes are connected by an edge, which means their feature similarities are above a threshold β.

Consider a set of patients, denoted by $P=\{p_{1},p_{2},\ldots ,p_{N}\}$, where each patient is denoted as $p_{i}$ for i={1,2,…,N}, with N being the total number of patients. Each patient, $p_{i}$, has an associated set of hospital stays, represented by $B_{i}=\left\{b_{i1},b_{i2},\ldots ,b_{iM_{i}}\right\}$. Here, $M_{i}$ denotes the total number of hospital stays for patient $p_{i}$. Each hospital stay $b_{ij}$, where *j* indexes the individual hospital stay for patient $p_{i}$, contains a set of medical notes. A sample patient knowledge graph is shown in Figure 4.

To analyse each patient’s medical record comprehensively per hospital stay, we aggregate all the medical notes of each hospital stay for that patient. This aggregated set of medical notes for patient $p_{i}$ for the *j*th stay is denoted by $C_{ij}$, which is defined in Equation (1) as the union of the medical notes from each hospital stay where *z* denotes each medical notes and *k* denotes the total number of medical notes during the *j*th hospital stay.

(1) $$C_{ij}=\overset{k}{\underset{z=1}{⋃}}c_{ij_{z}}$$

Let $S_{i}$ represent the set of all medical notes for patient $p_{i}$, combining data from all hospital stays as in Equation (2)

(2) $$S_{i}=\left\{C_{i1},C_{i2},\ldots ,C_{iM_{i}}\right\}$$

The primary purpose is to learn the model’s prediction $F_{\theta }$: $S\to {\hat{Y}}_{ij}$, where ${\hat{Y}}_{ij}$ represents the predicted likelihood for the target label $Y_{ij}$. Learnable modelling characteristics are denoted by θ.

**Figure 5:**
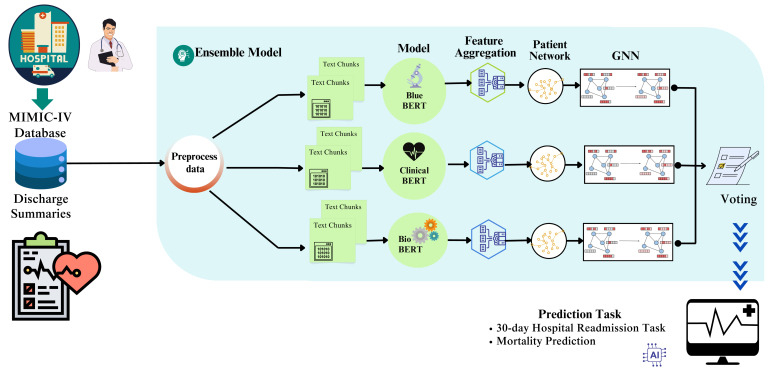
Overall architecture of the proposed PKGNN.

Figure 5 depicts the overall architecture of the proposed PKGNN.

### 3.3. Medical Notes Representation and Knowledge Graph

The observed medical notes $S_{i}$ for each patient are pre-processed to extract relevant information and create vectors that are used for graph node feature embeddings. Algorithm 1 describes the medical notes’ latent representation process based on feature aggregation for each patient’s hospital stay data. We utilise a pre-trained BERT variant model to tokenise medical notes $S_{i}$ and generate feature embeddings $d_{ij}=\left\{d_{ij}^{1},d_{ij}^{2},\ldots ,d_{ij}^{x_{j}}\right\}$, which represents the medical notes embedding vectors of size 768 for hospital stay *j*, as explained in Algorithm 1.
**Algorithm 1** Medical Notes Latent Representation1:**Input:** Medical notes $S_{i}$, for patient $p_{i}$.2:**Output:** Medical Notes Latent Representation3:            $D={⋃}_{i=1}^{N}\left\{d_{ij}|j\in \left\{1,2,\ldots ,M_{i}\right\}\right\}$4:**for** each i∈{1,…,N} **do**5:    **for** each hospital stay $j\in \{1,\ldots ,M_{i}\}$ **do**6:        Concatenate all the medical notes of *j*th hospital stay using$C_{ij}={⋃}_{z=1}^{k}c_{ij_{z}}$7:        Divide $C_{ij}$ into $x_{j}$ 512-byte chunks since the BERT model can only process 512 input sequences at once. $C_{ij}=\left\{c_{ij}^{1},c_{ij}^{2},\ldots ,c_{ij}^{x_{j}}\right\}$8:        **for** each chunk $c_{ij}^{x}$ where $x\in \{1,\ldots ,x_{j}\}$ **do**:9:           $a_{ij}^{x}=\text{BERT-Tokenizer}(c_{ij}^{x})$10:           $d_{ij}^{x}=\mathrm{BERT}\;\mathrm{variant}\;\mathrm{feature}\;\mathrm{vector}\left(a_{ij}^{x}\right)$11:        **end for**12:        Use the average feature aggregator to obtain the feature vector13:        $d_{ij}=\frac{1}{x_{j}}\sum_{x=1}^{x_{j}}d_{ij}^{x}$.14:    **end for**15:**end for**16:Note: $d_{ij}^{x}$ is of the dimension $768\times x_{j}$ (where 768 is the dimensionality of the BERT embeddings for each chunk), while $d_{ij}$ is of the dimension 768×1 (averaged feature aggregated vector).

For each hospital stay, the medical notes are concatenated and divided into 512-byte chunks, as the BERT model can only process sequences of this length. Each chunk $c_{ij}^{x}$ is tokenised using the BERT tokeniser, and a feature vector $d_{ij}^{x}$ is extracted using a BERT variant.

To obtain a single feature vector $d_{ij}$ for each hospital stay, the algorithm averages the feature vectors of all chunks. The resulting vector $d_{ij}$ has a dimensionality of 768×1, representing the averaged feature-aggregated vector. This process is repeated for all hospital stays, ensuring that each set of medical notes is transformed into a compact, meaningful representation suitable for further analysis or modelling tasks. This representation serves as a compressed latent encoding of the textual information within the medical notes, facilitating downstream predictive modelling tasks.

An undirected, unweighted knowledge graph *G* = (V,E) is constructed where *V* is the set of nodes and *E* is the set of edges. The set of nodes *V* is defined as follows:

(3) $$V=\overset{N}{\underset{i=1}{⋃}}\left\{b_{ij}|j\in \left\{1,2,\ldots ,M_{i}\right\}\right\}$$

The hospital stay of patient $p_{i}$ during their *j*-th visit is represented by $b_{ij}$, where *j* ranges from 1 to $M_{i}$. Here, the total number of hospital stays for all the patients are denoted by $M=\{M_{1},M_{2},\ldots ,M_{N}\}$, where $M_{i}$ represents the number of hospital stays for patient $p_{i}$.

Two nodes $b_{ip}$ and $b_{jq}$ corresponding to two hospital stays *p*, *q* are connected by an edge if their feature similarities are above a threshold β. The similarity score $l_{pq}$ for vertices is calculated using Equation (4).

(4) $$l_{pq}=1\;\mathrm{if}\;\mathrm{similarity}\left(d_{ip},d_{jq}\right)=\frac{d_{ip}\;\cdot \;d_{jq}}{∥d_{ip}∥∥d_{jq}∥}>\beta$$

where $l_{pq}$ represents an edge between hospital stay nodes with *p* and *q* corresponding to hospital stays, while *i* and *j* denote patients. Following hyper-parameter tuning, we set β = 0.95, as the average node similarity is high. This will help to link nodes with substantial similarity and reduce the occurrence of false positive predictions.

Here, the constructed knowledge graph is trained using a two-layer GCN model. Let the symmetric adjacency matrix of the graph be $L=\left[l_{ij}\right]\in R^{M\times M}$, where *M* is the size of the node set *V*. The corresponding degree matrix is represented by *T*, where $T_{ii}=\sum_{j}L_{ij}$. The adjacency matrix *L* is augmented with self-loops to form $\tilde{L}=L+I_{M}$, where $I_{M}$ is the identity matrix.

The normalised adjacency matrix $\hat{L}$ is computed as follows:

(5) $$\hat{L}={\tilde{T}}^{-1/2}\tilde{L}{\tilde{T}}^{-1/2}$$

where $\tilde{L}=L+I_{M}$ is the adjacency matrix augmented with self-loops, and $\tilde{T}$ is the degree matrix of $\tilde{L}$, with ${\tilde{T}}_{ii}=\sum_{j}{\tilde{L}}_{ij}$.

In Equation (5), the matrix $\hat{L}$ is used in the GCN to aggregate information from node *i* and its neighboring nodes, with normalisation based on the degrees of the nodes. Specifically, the feature representation of each node is updated by combining its own features with those of its neighbors, weighted by the normalised adjacency matrix. For an undirected and unweighted graph, this weighting is based on the degrees of the nodes, ensuring that the contributions of neighbors are balanced. This process is formalised in the update rule for the *g*-th layer:

(6) $$H^{\left(g+1\right)}=\sigma \left(\hat{L}H^{\left(g\right)}w^{\left(g\right)}\right)$$

where $w^{\left(g\right)}$ is the trainable weight matrix, σ indicates the ReLU activation function, which is applied element-wise to induce non-linearity, and $H^{\left(g+1\right)}$ is the matrix of activations in the *g*-th layer.

**Figure 6:**
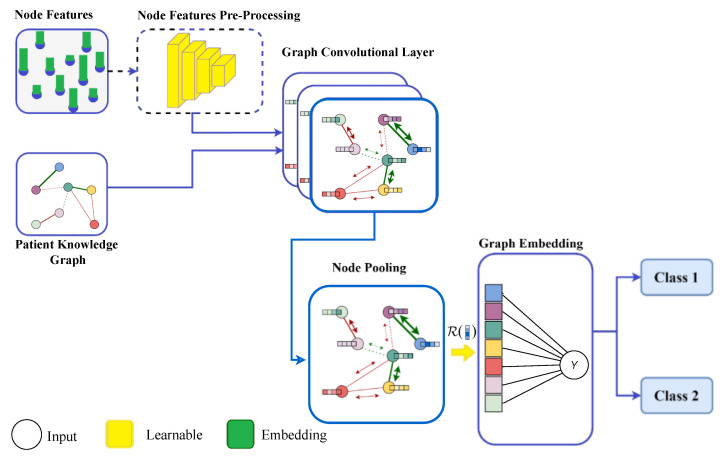
The framework of a classification model using a graph convolutional network. Node characteristics and a patient knowledge graph structure make up the model’s input. After that, pre-processing may be applied to the node features. After that, a block of graph convolutional layers receives the node characteristics and uses them to learn node embeddings. The graph embeddings are then learnt using a node pooling module. Finally, the results are predicted using the graph embeddings.

To perform classification, the softmax function (Equation (7)) is applied to the forward model in Equation (5) to obtain class probabilities:

(7) $$Y=\mathrm{softmax}\left(\hat{L}\;\mathrm{ReLU}\left(\hat{L}Xw^{\left(0\right)}\right)w^{\left(1\right)}\right)$$

where *X* is the matrix of node feature embeddings and $w^{\left(0\right)}$ and $w^{\left(1\right)}$ are the input-to-hidden and hidden-to-output weight matrices for the two-layer GCN, respectively. A GCN model for classification is illustrated in Figure 6.

### 3.4. Loss Function

Cross-entropy loss is frequently utilised when outcomes are categorised, for instance, in clinical risk classification. The cross-entropy loss function $L_{\mathrm{CE}}$ for all labelled examples is expressed in Equation (8), as follows:

(8) $$L_{\mathrm{CE}}=-\sum_{i\in M}\sum_{j=1}^{Q}Y_{ij}\ln\hat{Y_{ij}}$$

where *M* is the set of indices of labelled vertices in the graph, and *Q* is the output feature dimension, equal to the number of classes. And $Y\in R^{|M|\times Q}$ is the label indicator matrix.

### 3.5. Ensemble Graph Learning

The proposed ensemble model uses three BERT variants: Clinical BERT [9], Bio BERT [10], and Blue BERT [11]. These models individually extract medical notes’ feature representations and create patients’ hospital stay feature vectors. The proposed ensemble model uses an aggregator to generate a fixed feature vector of medical notes on top of the BERT variants. This technique captures extended word interdependence, which is essential in clinical situations.

Algorithm 2 describes the whole working procedure of the proposed PKGNN framework.
**Algorithm 2** Proposed PKGNN framework1:Initialisation:  ←  Learning_rate, Batch_size, seed, max_grad_norm,GCN: Input_size, hidden_size, out_size, num_layers, and threshold2:Obtain the feature aggregated embedding $d_{ij}$ from Algorithm 1.3:**for** each classifier ${\left\{F_{j}\right\}}_{j=1}^{3}$ **do**4:    **for** each epoch **do**5:        Build a graph G, with node features and edge connections based on the cosine similarity of node embeddings using Equation (4)6:        Train the GCN model with the node features.7:        Calculate the binary cross-entropy loss using Equation (8)8:        Update parameters using Adam optimiser9:    **end for**10:**end for**11:Use ensemble approach to obtain $F_{\mathrm{ev}}\left(X\right)$ the predictions using majority voting Equation (9).12:Test and validate the trained model predicting the probability scores.

Here, we consider three classifiers based on Clinical BERT, BioBERT, and BlueBERT, respectively. Let $F_{1},F_{2}$, and $F_{3}$ denote the three classifiers. The ensemble voting classifier $F_{\mathrm{ev}}$ predicts the class ${\hat{y}}_{e}$ from the predictive score of the individual classifiers. The trained GCN models are integrated into the ensemble model. The majority voting classifier determines the final output of the ensemble method, which aggregates the predictions of the three classifiers, as described in Equation (9):

(9) $$F_{\mathrm{ev}}\left(X\right)=\arg\max\sum_{e=1}^{3}I\left(C_{e}\left(S\right)={\hat{Y}}_{e}\right)$$

In this equation, $C_{e}\left(S\right)$ represents the prediction of the $e^{th}$ classifier for input *S*. The indicator function is I(·), which returns 1 if the argument is true and 0 otherwise. The term $\sum_{e=1}^{3}I\left(C_{e}\left(S\right)={\hat{Y}}_{e}\right)$ counts the number of classifiers that predicted class ${\hat{Y}}_{e}$.

This study demonstrates that the proposed ensemble graph-based learning approach (PKGNN) is a valuable technique for enhancing the performance of clinical prediction models, in contrast to most previous attempts to construct $F_{\theta }$.

## 4. Results

We implemented the code with python 3.12.4, pytorch-cuda 11.7, and trained all the models on a workstation with Intel ^®^ Xeon^TM^ processor (Intel, Santa Clara, CA, USA), NVIDIA Quadro P5000 Graphics Card, 64 GB RAM (NVIDIA, Santa Clara, CA, USA).

### 4.1. Evaluation Metrics

The outcome of this classification must be assessed and quantified to determine whether or not the samples are correctly categorised. Accuracy, precision, recall, and AUROC are used as evaluation metrics.

True Positive (TP): Instances of deceased patients that were correctly identified as deceased.

False Positive (FP): Instances of survived patients that were misclassified as deceased.

True Negative (TN): Survived patients’ instances that were correctly identified as survived.

False Negative (FN): Deceased patients’ instances that were misclassified as survived.

*Precision*: Precision measures how many positive predictions are correct. The precision of a model is 1.0 if it generates no false positives. The formula is as follows:

(10) $$Precision=\frac{TP}{TP+FP}$$

*Recall*: The capacity to recognise each relevant value in the data collection is known as recall.

(11) $$Recall=\frac{TP}{TP+FN}$$

*Accuracy*: Accuracy describes the number of correct and overall predictions.

(12) $$Accuracy\;=\frac{TP+TN}{\;\mathrm{Number}\;\mathrm{of}\;\mathrm{test}\;\mathrm{samples}\;}$$

*AUROC*: The area under the ROC curve is the AUROC for a particular curve. The best AUROC is 1, while the lowest is 0.5. The trade-off between TP and FP at various decision thresholds between 1 and 0 is displayed by the AUROC curve. For unbalanced data, this measure provides extra information.

*AUPRC*: The Area Under the Precision-Recall Curve (AUPRC) is a metric used particularly in scenarios with imbalanced datasets. It summarises the trade-off between precision and recall across different classification thresholds. A higher AUPRC indicates better model performance, with a maximum value of 1 representing perfect precision-recall balance.

*R@P80*: Recall at 80% precision, indicating the recall when the precision is fixed at 80%. The formula can be expressed as follows:

(13) R@P80=max{Recall|Precision≥0.80}

### 4.2. Patient Knowledge Graph Framework

We comprehensively evaluate and compare the proposed method against six state-of-the-art (SOTA) methods. Table 1 shows that the PKGNN model achieves better performance than state-of-the-art results, where ensemble learning for a global patient graph with a feature aggregation method improves performance for 30-day hospital readmission prediction and mortality prediction. For the hospital readmission task, the proposed model achieves an AUROC of 0.951 and an AUPRC of 0.754, surpassing all competing models. Likewise, it attains an AUROC of 0.934 and an AUPRC of 0.652 for mortality prediction, consistently outperforming all competing models.

The model has been trained to minimise loss using the ensemble learning method with a binary cross-entropy loss function. The training configuration is set with a random seed of 42 to ensure the reproducibility of results. The model has been trained for 100 epochs, with logging occurring every 1000 iterations, validation after each epoch, and model checkpoints saved every 10 epochs. To prevent exploding gradients, gradient clipping is applied with a maximum gradient norm of 100. The batch size for training is set to 32. The optimiser is Adam, with a learning rate of 0.01, weight decay of 0.0005, and beta values of 0.9 and 0.999 for the first- and second-moment estimates, respectively. A step learning rate scheduler is employed, which reduces the learning rate by gamma (1.0) every 100 steps.

The dataset configuration specifies a graph-based dataset stored at the root path and uses a threshold of 0.99 for data processing. The GCN model with ensemble learning has an input feature size of 768, a hidden layer size of 16, and an output size of 2, indicating a binary classification task. The GCN has two layers and includes dropout with a probability of 0.5 to prevent over-fitting.

**Figure 7:**
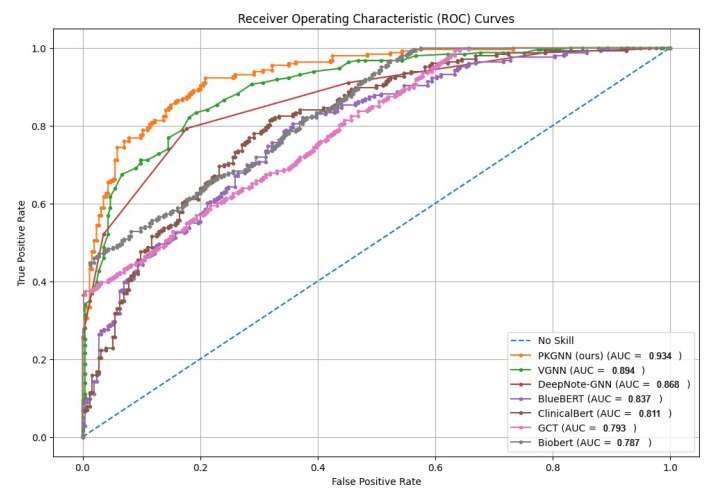
AUROC for in-hospital mortality prediction.

**Figure 8:**
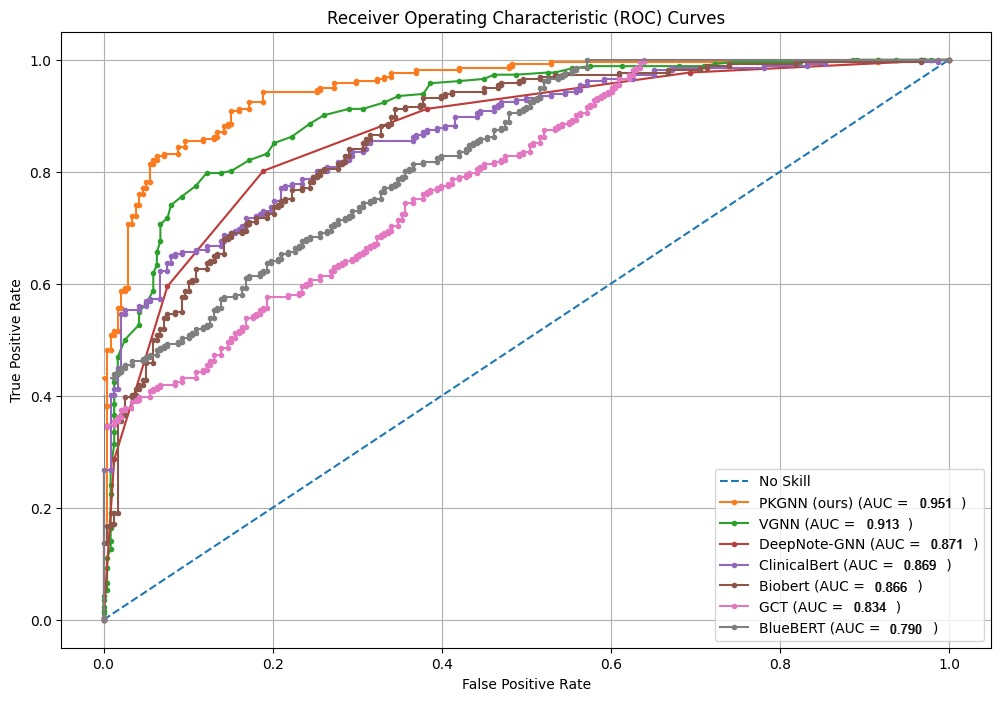
AUROC for 30-day hospital readmission prediction.

Figure 7 and Figure 8 show the comparative AUROC plot for mortality prediction and 30-day hospital readmission.

### 4.3. Ablation Study

In Table 2 and Table 3, we performed ablation tests to analyse the efficacy of the predicted task and relationship ensemble module and global patient graph module. In the MIMIC-IV dataset’s prediction task, we compare these experiments.

For the hospital readmission task, Set 1 includes 42,671 nodes and 472,435,459 edges, achieving the highest performance with an AUROC of 0.955, an AUPRC of 0.754, and a recall at 80% precision (R@P80) of 0.641. Set 2, with 6,162 nodes and 7,456,808 edges, shows a slight decrease in performance with an AUROC of 0.934, an AUPRC of 0.652, and an R@P80 of 0.575. Set 3, the smallest set, comprises 3700 nodes and 3,591,982 edges, resulting in an AUROC of 0.903, an AUPRC of 0.604, and an R@P80 of 0.455.

For the mortality prediction task, we again evaluated three sets. Set 1, the largest, includes 42,671 nodes and 472,435,459 edges, achieving an AUROC of 0.934, an AUPRC of 0.652, and an R@P80 of 0.575. Set 2, with 15,292 nodes and 59,643,760 edges, shows an AUROC of 0.917, an AUPRC of 0.544, and an R@P80 of 0.515. Set 3, which contains 6162 nodes and 7,456,808 edges, has the lowest performance, with an AUROC of 0.899, an AUPRC of 0.541, and an R@P80 of 0.415.

These results indicate that more extensive sets of nodes and edges generally improve the predictive performance of the proposed models for both hospital readmission and mortality prediction tasks. The significant performance drop in smaller sets highlights the importance of comprehensive data inclusion in constructing patient graphs.

## 5. Discussion

Deep learning algorithms for analysing raw health data in ICUs have tremendous potential for improving patient outcomes. These advanced methods enable real-time analysis of complex and unstructured data, facilitating the rapid identification of essential patterns, predicting patient deterioration, and supporting clinicians in their decision-making. Thus, we propose an ensemble patient graph framework with BERT variants: Clinical BERT, BioBERT, and BlueBERT were leveraged as cutting-edge natural language processing models pre-trained on healthcare-specific datasets. These models provide context-specific word representations from medical notes, enhancing generalisation capability and capturing the extended dependencies between words, which are crucial in clinical settings.

We successfully developed the PKGNN framework, a promising and ensemble GCN-based approach to address clinical and biomedical information complexities. The framework provides a structured and meaningful representation of clinical and biomedical data by constructing knowledge graphs and applying an ensemble approach. The ensemble model aims to leverage the strengths of both models to improve overall performance in predicting patient fatality. The ensemble approach employed in the framework excels at uncovering latent patterns and associations within the data. This capability can reveal critical insights that may have otherwise remained hidden. The performance evaluation on the MIMIC-IV dataset demonstrates that PKGNN outperforms the state-of-the-art baselines across two different tasks: mortality prediction and 30-day hospital readmission prediction.

The current study focuses on mortality prediction and 30-day hospital readmission, but there are other critical clinical outcomes that could benefit from similar predictive modelling. Future work could expand the framework to predict disease progression, treatment response, and other relevant clinical outcomes, providing a more comprehensive approach to patient risk assessment.

As technology and data collection methods in healthcare continue to evolve, ongoing research should also investigate the integration of additional data sources, such as genomic data or real-time sensor data from wearable devices, to further enhance the model’s predictive capabilities.

## 6. Conclusions

This study highlights the importance of integrating the graphical structure of EHR data to enhance predictive performance in critical healthcare tasks such as mortality and 30-day hospital readmission prediction. By leveraging advanced NLP models like Clinical BERT, BioBERT, and BlueBERT for medical note extraction and incorporating them into a patient knowledge graph, the proposed PKGNN framework effectively captures meaningful clinical relationships. The model’s performance metrics on the MIMIC-IV dataset demonstrate that utilising patient graph structures significantly improves prediction accuracy. This work contributes to the literature by advancing strategies for knowledge graph-based EHR analysis, offering a robust approach to risk prediction and supporting more informed clinical decision-making.

## Figures and Tables

**Table 1:** AUROC for 30-day hospital readmission and mortality prediction.

Model	Hospital Readmission		Mortality Prediction	
	AUROC	AUPRC	AUROC	AUPRC
BlueBERT [11]	0.790	0.512	0.787	0.461
GCT [7]	0.834	0.581	0.793	0.582
Biobert [10]	0.866	0.570	0.811	0.574
Clinical BERT [9]	0.869	0.612	0.837	0.602
DeepNote-GNN [20]	0.871	0.653	0.868	0.613
VGNN [30]	0.913	0.624	0.894	0.634
PKGNN (proposed)	0.951	0.754	0.934	0.652

**Table 2:** The 30-day hospital readmission prediction.

			AUROC	AUPRC	R@P80
Set 1	nodes	42,671	0.951	0.754	0.641
	edges	472,435,459			
Set 2	nodes	6162	0.934	0.652	0.575
	edges	7,456,808			
Set 3	nodes	3700	0.903	0.604	0.455
	edges	3,591,982			

**Table 3:** In-hospital mortality prediction.

			AUROC	AUPRC	R@P80
Set 1	nodes	42,671	0.934	0.652	0.575
	edges	472,435,459			
Set 2	nodes	15,292	0.917	0.544	0.515
	edges	59,643,760			
Set 3	nodes	6162	0.899	0.541	0.415
	edges	7,456,808			

## Data Availability

The data underlying the results presented in the work are available from the MIMIC-IV, an extensive, single-centre database comprising information about patients admitted to critical care units at a large tertiary care hospital. More details about MIMIC-III can be found on their website (https://mimic.mit.edu/about/mimic/ accessed on 3 February 2025). To access these data, interested researchers must complete the CITI ’Data or Specimens Only Research’ course (https://www.citiprogram.org/index.cfm?pageID=154&icat=0&ac=0) accessed on 3 February 2025 and then apply for credentialed access through PhysioNet (https://physionet.org/content/mimiciv/) accessed on 3 February 2025.
